# Mental health clothing bank - adDressing the issue

**DOI:** 10.1192/bjo.2021.604

**Published:** 2021-06-18

**Authors:** Louisa Ward

**Affiliations:** Worcestershire health and care NHS trust

## Abstract

**Aims:**

We often have patients who are admitted to the ward wearing only the clothes they came in. These patients have no way of going to get more clothes due to being detained, poverty/ homelessness or covid restrictions. Many do not have friends or family who can bring them clothes. As such they might wear one set of clothes for a number of weeks which is bad for their physical and mental health. We are creating a clothes bank to provide a change of clothes for these patients, and help their recovery back into the community. Many have clothes that are inappropriate for the current weather, or do not have a set of smart enough clothes for a job interview. We feel that this simple intervention will have a big community impact.

**Method:**

We have obtained support from a number of charities and companies to supply donations. The project will be led by a team of staff and patients.

**Result:**

We will review the usage of this scheme in 6 months time

**Conclusion:**

We hope this intervention will tackle the issue of clothing on mental health wards. In the future we wish to expand this to outpatient mental health service users. We would then like to expand this project countrywide as are unaware of any other areas providing something similar.

